# Treatment with YIGSR peptide ameliorates mouse tail lymphedema by 67 kDa laminin receptor (67LR)-dependent cell-cell adhesion

**DOI:** 10.1016/j.bbrep.2023.101514

**Published:** 2023-07-20

**Authors:** Y. Sakae, H. Takada, S. Ichinose, M. Nakajima, A. Sakai, R. Ogawa

**Affiliations:** a Department of Plastic, Reconstructive and Aesthetic Surgery, Nippon Medical School, Japan; b Department of Anti-Aging and Preventive Medicine, Nippon Medical School, Japan; c Department of Pharmacology, Nippon Medical School, Japan

**Keywords:** Lymphedema, YIGSR peptide67, kDa laminin receptor, Cell-cell adhesion, Extracellular matrix

## Abstract

Impaired microcirculation can cause lymphatic leakage which leads to a chronic swelling in the tissues of the body. However, no successful treatment gives any protection against lymphedema due to the lack of well-revealed pathophysiology of secondary lymphedema. Binary image of laminin immunohistochemical expression revealed that distribution of laminin expression localized during surgically induced lymphedema. 67 kDa laminin receptor (67LR) mRNA expression showed a peak at during lymphedema exacerbation. Since the response of 67LR molecules may affect the prevention of inflammation and edema, here we have hypothesized that 67LR ligand of YIGSR peptide could permit reconstructive environment for amelioration of lymphedema and evaluated the effect of YIGSR in a mouse tail model of lymphedema. Indeed, intra-abdominal injections of YIGSR for the first 3 days after inducing lymphedema in the mouse tail model reduced the tail lymphedema on day 14 by 27% (P = 0.035). Histology showed that YIGSR treatment protected lymphedema impairment in epidermis and dermis, and it also inhibited the expansion of intercellular spaces and enhanced especially cell adhesion in the basement membrane as revealed by transmission electron microscopy. Interestingly, the treatment also reduced the local expression of transforming growth factor (TGF)β. Further elucidation of the mechanisms of 67LR-facilitated lymphangiogenesis contributes to find potential targets for the treatment of lymphedema.

## Highlights

• Laminin peptide YIGSR-NH_2_ may create a permissive environment for lymphedema amelioration.
• 67 kDa laminin receptor is useful as a marker for sustained inflammation and tissue remodeling.
• YIGSR-NH_2_ reduced the expression of TGFβ and enhanced cell-cell adhesion to restore epidermis and vessel barrier function.

## 1 Introduction

The microcirculation not only delivers oxygen and nutrients to cells via arterioles and capillaries, it also removes the cellular waste products and excess interstitial fluid via the lymphatic system. Therefore, when the lymphatic system is impaired, debilitating chronic swelling of the tissues of the body can occur. This is due not only to fluid accumulation but also chronic and progressive inflammation that promotes fat deposition. The inflammation also generates transforming growth factor (TGF)-β, which induces fibrosis of the lymphatics. This promotes poor wound healing, recurrent infections, and occasionally secondary malignancies [1]. The inflammation may also promote hyperkeratosis, a common feature of advanced lymphedema [2]. The causes of lymphedema can be congenital abnormalities or secondary causes such as obstruction, damage, or infection of the lymphatic system. In particular, lymphedema secondary to radiotherapy and/or the surgical removal of lymph nodes for cancer control is a common and growing problem globally. However, an established curative treatment for secondary lymphedema has yet to be found, largely because the research on the lymphatic system has fallen behind that on blood vessels. As a result, methods that successfully prevent, ameliorate, or cure secondary lymphedema are lacking [[3], [4], [5], [6], [7], [8], [9], [10]].

Of particular interest to our study, the basement membrane and specifically laminin which consists of α, β, and γ heterotrimeric chains. Laminin is a member of the cell adhesion proteins, which together with other macromolecules (i.e. structural proteins and glycosaminoglycans) form the extracellular matrix (ECM). In particular, laminin is essential for the complex functions of the basement membrane, which include acting as a mechanosensitive cellular anchorage site, a physical barrier, and a collector of signals [11]. Laminin play a key role in the junctional tightness between endothelial cells and the resulting vascular impermeability [12]. Without laminin β chain, laminin can neither accumulate nor basement membrane can form [13]. Recently, it has been suggested that regulation of the expression and function of the 67-kDa laminin receptor (67LR), a non-integrin cell membrane receptor that binds laminin β, is one of the key factors for the treatment of vasogenic edema formation [14]. Blockade of 67LR function has been suggested to evoke the increase of vascular permeability [15] and concomitantly cause upregulation of laminin expression [14]. 67LR activation may be one of the therapeutic strategies to reduce vascular permeability [16,17]. Notably, the binding of 67LR to laminin β is mediated by the YIGSR sequence [18], which occurs in a cysteine-rich site of the laminin β short arm [19]. Thus, these studies together suggest that the 67LR-laminin β interaction mediated by YIGSR may attenuate lymphedema by promoting basement membrane repair and cell-cell adhesion, and that YIGSR peptide might have therapeutic benefits in this setting.

To test these hypotheses, we injected mice intra-abdominally with YIGSR peptide shortly after initiating lymphedema in the mouse tail lymphedema model, which is a well-known model of secondary lymphedema that is generated by ablating the collecting lymphatic vessels in the tail. We also blocked 67LR in some mice via intra-abdominal injections of MLuC5, an anti-67LR antibody [11]. The effect of these injections on the lymphedema and the morphology of the lymphatic vessels in the tail was assessed by measuring the tail diameter, histology, immunohistochemistry, transmission electron microscopy (TEM), and reverse transcription-quantitative PCR (RT-qPCR) during the course of the lymphedema.

## 2 Material and methods

### 2.1 Animals

All animal experiments were performed according to the guidelines prescribed by the Animal Care and Use Committee of Nippon Medical School. The protocol in this study was approved by the Animal Experiments Ethical Review Committee of Nippon Medical School (approval number 2022–030). Female Slc ICR mice (6–8 week-old; weight range 21–27 g) (Sankyo Labo Service Corp., Inc.; Japan) were used in all animal experiments. Up to four mice were housed per cage. The mice were allowed to adapt to their vivarium for 1 week before experiments began. In total, 437 mice were used in this study.

### 2.2 Mouse tail lymphedema model

**Fig. 1:**
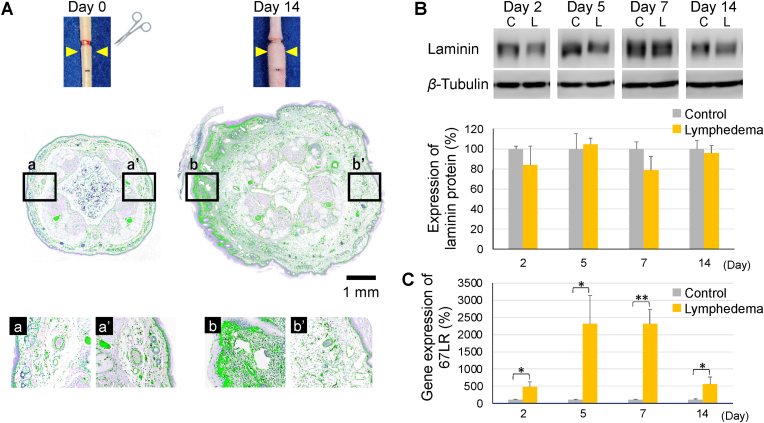
Laminin localization and 67LR expression rises during the development of lymphedema. (A) Secondary lymphedema in the mouse tail (above). Representative tail cross-sections of lymphedema mice and binary images of immunohistochemical expression of laminin (second and third row). (B) Laminin protein expression measured by Western Blotting (n = 3–6). (C) RT-qPCR of 67LR expression in tail tissues from untreated operated and non-operated mice (n = 6). Similarly, RT-qPCR data of Prox1 and Lyve1 (biomarkers of lymphatic endothelial cells) expression in tail tissues are shown in Supplementary Figure S1. Mean ± SEM are shown in the plots. *P < 0.03, **P < 0.001, as determined by Student's one-sided *t*-test comparing operated mice to non-operated mice (n = 6).

Tail lymphedema was induced in mice as described previously [[3], [4], [5], [6], [7], [8], [9], [10]]. Briefly, mice were anesthetized with 2.5% isoflurane, after which a 2 mm-wide strip of full-thickness tail skin that ran the circumference of the tail was removed 2-cm distal from the tail base (Fig. 1A, above). The collecting lymphatic vessels on both sides of the lateral tail veins were then ablated.

### 2.3 Immunohistochemical and quantification of laminin expression

For immunohistochemical, the whole tails were frozen, after which 10 μm-thick cross-sections were cut, fixed with 4% paraformaldehyde, deparaffinized, washed with Tris-buffered saline (TBS), fixed with 1% hydrogen peroxide and methanol, washed again with TBS, blocked with skim milk, and incubated overnight at 4 °C with polyclonal rabbit anti-mouse laminin IgG (1:500; Abcam: ab11575) antibody. The sections were then washed with TBS, incubated with the Histofine simple stain mouse MAX-PO (R) (Nichirei Bioscience; Tokyo, Japan) secondary antibody, and subsequently incubated with DAB substrate (Takara Bio). Counter staining was performed with Mayer's hematoxylin. The data were analyzed with OlyVIA software (Olympus; Tokyo, Japan). The laminin expression in the immunohistochemical sections was quantified by a semiautomated binarization process using WinROOF2021 (Mitani Corp.; Fukui/Tokyo, Japan) software (Fig. 1A, second and third row).

### 2.4 Immunoblotting

Tails were homogenized in 10 mM Tris–HCl (pH 7.2) containing 250 mM sucrose, 10 mM HEPES, 10 mM EDTA and protease inhibitor cocktail (Merck, Darmstadt, Germany). Homogenates were centrifuged at 12000 rpm, at 4 °C for 20 min and the supernatants were collected. Next, samples were electrophoresed on 5% (for laminin) or 10% (for β-tubulin) SDS-polyacrylamide gels and electroblotted onto PVDF membranes (GE Healthcare, Chicago, IL). After blocking, membranes were incubated with an anti-laminin (1:1000; ab11575, Abcam, Cambridge, UK) or an anti-*β*-tubulin (1:1000; 9F3, Cell Signaling Technology, Danvers, MA) antibody in PBS containing 0.1% Tween-20 at 4 °C overnight and then detected using an HRP-linked anti-rabbit IgG antibody (1:2000; #7074, Cell Signaling Technology) and chemiluminescence (ECL Prime Western Blotting Detection Reagents; GE Healthcare). The luminescence was detected with a C-DiGit Blot Scanner (LI-COR Biosciences, Lincoln, NE). Optical densities of bands were quantified using Image J (Fig. 1B).

### 2.5 Real-time reverse transcription-quantitative PCR (RT-qPCR)

On days 2, 5, 7 and 14, tail samples were excised and immediately frozen with liquid nitrogen. Total RNA was extracted using RNAiso Plus (Takara Bio, Shiga, Japan). RNA quantification was performed using NanoDrop One (Thermo Fisher Scientific, Waltham, MA). Total RNA (500 ng) was reverse-transcribed with a random primer using an iScript Select cDNA Synthesis kit (Bio-Rad Laboratories, Hercules, CA). Quantitative PCR was performed using Power SYBR Green Master Mix (Thermo Fisher Scientific). Primer sequences used in this study were shown in Supplementary Table S1. Relative expression was analyzed using the 2^−ΔΔCt^ method (Fig. 1, Fig. 4C).

### 2.6 Edema evaluation

**Fig. 2:**
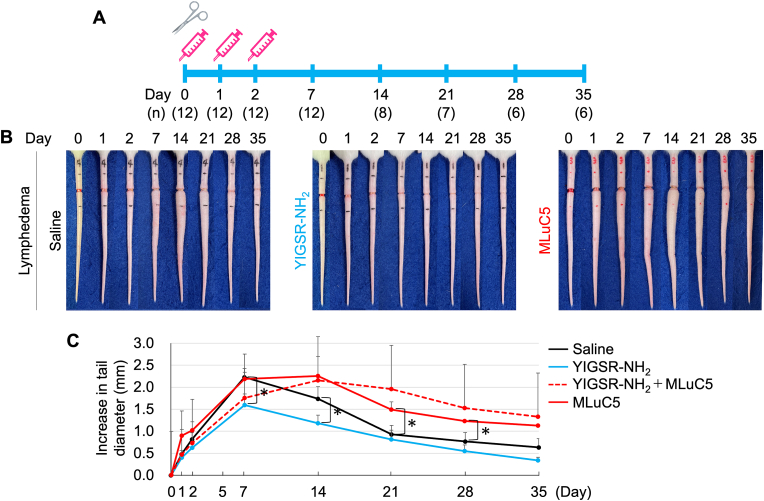
Effect of YIGSR injections on surgically induced lymphedema. (A) Schematic depiction of the treatment protocol. The scissor image indicates the operation makes lymphedema was conducted on Day 0. The syringe symbol image indicates when the mice were injected intra-abdominally with YIGSR, MLuC5, or saline as a control. The number of mice is shown in parentheses on the each timepoint. (B) Macroscopic view of the tail swelling that arose over time in the mouse tail lymphedema model. The tails on the left are from untreated operated mice while those on the right are from MLuC5-injected operated mice and middles are from YIGSR-injected operated mice. (C) Plot showing the effect of YIGSR, YIGSR + MLuC5, or MLuC5 treatment on tail edema over time. Mean ± SEM are shown in the plots (YIGSR, MLuC5: n = 6–12, YIGSR + MLuC5: n = 3). *P < 0.05 as determined by Student's one-sided *t*-test.

Immediately after lymphedema induction, the mice were intra-abdominally injected with 500 μl of YIGSR-NH_2_ (1 mg/mouse) saline solution or saline for 3 consecutive days starting on the day of lymphatic ablation (i.e. days 0, 1 and 2) (Fig. 2A). The dose of 1 mg/mouse is on the basis of the previous report of EGCG, ligand of 67LR [1]. Alternatively, the mice were injected in the same manner with MLuC5 (Santa Cruz Biotechnology, Inc., Dallas, TX), a monoclonal antibody against 67LR (100 μg/ml). Non-operated untreated mice served as controls for some experiments. Tail edema was determined on days 0, 1, 2, 7, 14, 21, 28, and 35 by using calipers (MA Corp.; Japan) to measure the tail diameter at the most edematous section, which lay approximately 2-mm distal of the incision (Fig. 2B and C).

### 2.7 Histology

On postoperative days 2, 5, 14 and 35, mouse tail tissues ∼2-mm distal of the incision were excised. For histology, the tissues were fixed with 4% paraformaldehyde, embedded in OCT compound (Sakura Finetek, Tokyo, Japan), and frozen with dry ice in acetone. Subsequently, 20 μm-thick cross-sections of the tail were cut from the frozen tissues, followed by drying in a dryer for 2 h and then staining with Masson's trichrome and hematoxylin and eosin (H&E). Masson's trichrome staining was utilized to quantify collagen fibers area.

### 2.8 TEM

Tail specimens obtained on days 2, 5, 14, and 35 were ﬁxed with 2.5% glutaraldehyde in 0.1 M phosphate buffer (PB) for 2 h, washed overnight at 4 °C in PB, post-ﬁxed with 1% OsO_4_ buffered with 0.1 M PB for 2 h, dehydrated in a graded series of ethanol, and embedded in Epon 812. Ultrathin cross-sections (80–90 nm thickness) were collected on copper grids, double-stained with uranyl acetate and lead citrate, and then examined by TEM (JEM-1400plus, JEOL; Tokyo, Japan).

### 2.9 Statistics

All experiments were performed independently at least twice with identical results. The quantitative macroscopic, immunohistochemical, histological, and RT-qPCR values were expressed as mean ± SEM. Groups were compared by using one-tailed Student's *t*-tests. *P* values < 0.05 were considered significant.

## 3 Results

### 3.1 Laminin can localize its deposition without altering total volume during the development of lymphedema, resulting in increased expression of 67LR

In the mouse tail lymphedema model, the collecting lymphatic vessels on both sides of the lateral tail vein are excised (Fig. 1A, above), thereby obstructing lymphatic flow and inducing acute lymphedema. Binary images of immunohistochemical expression of laminin revealed that the distribution of laminin expression localized to sites of prominent exacerbation (Fig. 1A and b) when edema peaked 14 days after this surgically induced lymphedema (Fig. 1A, homogeneous distribution: a vs a’, differences in local distribution: b vs b’). After the onset of lymphedema, there was no change in laminin protein expression compared to controls (Fig. 1B), while 67 kDa laminin receptor (67LR) mRNA expression peaked during exacerbation of lymphedema (Fig. 1C). Real-time qPCR of 67LR mRNA expression showed peaks at days 5 and 7, followed by a decline at day 14.

### 3.2 YIGSR treatment suppresses surgically induced lymphedema

To determine whether the 67LR ligand YIGSR can block lymphedema in the mouse tail edema model, the mice were injected intra-abdominally with the peptide for 3 consecutive days (Fig. 2A). Compared to saline-treated negative control mice, YIGSR treatment ameliorated the tail lymphedema (as shown by tail diameters) by 27% on day 7 (1.6 *vs.* 2.2 cm, *P* = 0.035). Similarly, on day 14, YIGSR treatment reduced the tail edema by 29% (1.2 *vs.* 1.7 cm, *P* < 0.05) (Fig. 2B and C). YIGSR activates 67LR by binding to it [2]. Since YIGSR treatment reduced mouse tail edema, we asked whether blocking 67LR activation with a specific antibody could exacerbate mouse tail edema. Indeed, intra-abdominal injection with the anti-67LR antibody MLuC5 [11] both significantly increased the lymphedema and prolonged the lymphedematous state (Fig. 2B and C). Moreover, intraperitoneal injection of YIGSR-NH_2_ and MLuC5 in mice caused tail swelling more slowly and delayed peak edema by one week compared to administration of MLuC5 alone (dashed red line as shown in Fig. 2C). No abnormalities were observed in mice for 35 days after intraperitoneal injection of YIGSR-NH_2_.

### 3.3 YIGSR treatment reduces the lymphedema-associated histological changes

**Fig. 3:**
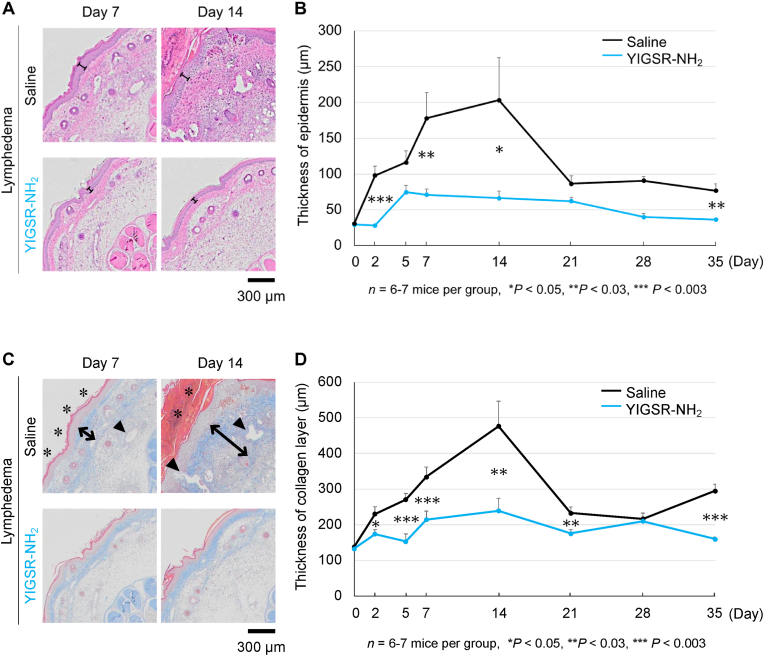
Effect of YIGSR injections on lymphedematous tail histology. (A, B) Effect of YIGSR on epidermal thickness. (A) Shows representative histological images (H&E staining). Epidermal thickness is shown by the bar. The plot in (B) shows the change in epidermal thickness over time. Mean ± SEM are shown in the plots (n = 6 or 7). *P < 0.05, **P < 0.03, ***P < 0.003, as determined by Student's *t-*test. (C) Representative tail cross-sections of the YIGSR- or saline-treated lymphedema mice (Masson's Trichrome staining). The double arrow lines indicate the thickness of edematous tissues. The arrowheads indicate the lymphatic vessel dilation. The asterisks indicate the hyperkeratosis. The plot in (D) shows the change in thickness of collagen layer in the edematous granulated tissue over time. Mean ± SEM are shown in the plots (n = 6 or 7). *P < 0.05, **P < 0.03, ***P < 0.003, as determined by Student's *t-*test.

The effect of YIGSR injection on the thickness of the epidermis at the most edematous section of the tail was assessed by H&E staining and low-power photographs. The injection greatly decreased the epidermal thickness compared to day 0 by 80% (35.6 vs 173.9 μm, *P* < 0.05, day 14) (Fig. 3A and B).

**Fig. 4:**
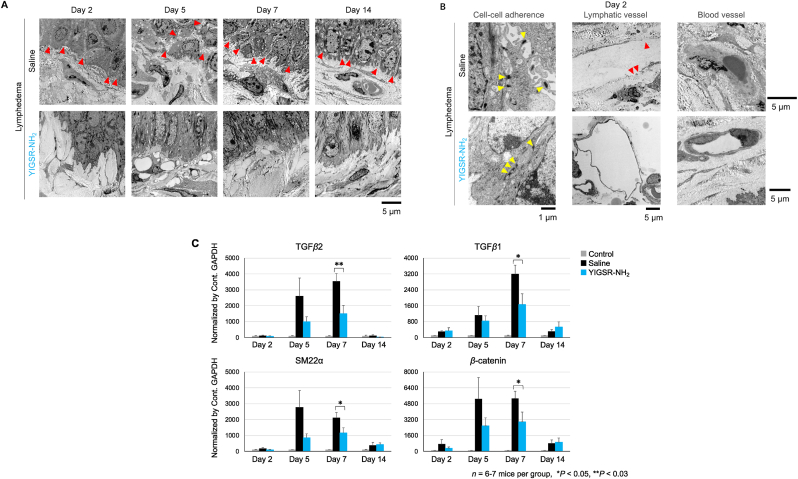
TEM images of the tails of saline- and YIGSR-treated lymphedema mice and the effect of YIGSR treatment on TGFβ1, TGFβ2, SM22α and β-catenin mRNA expression. (A) TEM studies revealed morphologically, cell-cell adhesion at basal membrane in the saline-treated lymphedema differed significantly from cells contained in the YIGSR-treated lymphedema. Red arrowheads indicate intercellular spaces between keratinocytes. (B) Representative images showing keratinocyte cell-cell adhesion. Yellow arrowheads indicate cell-cell adherence. Red arrowheads indicate damaged sites in lymphatic endothelial cell. In blood vessels, in control the nucleus of vascular endothelial cell was degenerated and the structure of vessel was damaged, but in YIGSR treated group, the structure of vessel and the nucleus of endothelial cell were sustained. (C) The plots show the mRNA expression of the indicated genes normalized by GAPDH expression, as measured by RT-qPCR. The data are shown as mean ± SEM (n = 6 or 7). *P < 0.05, **P < 0.03, as determined by Student's *t*-test comparing YIGSR to saline. (For interpretation of the references to colour in this figure legend, the reader is referred to the Web version of this article.)

As shown by low-power photographs of the Masson's trichrome-stained tails (see Supplementary) and high-power photographs of the inset areas (Fig. 3C), YIGSR treatment markedly ameliorated the lymphatic vessel and dermal architectural changes that were induced by the lymphatic ablation. Specifically, the treatment sustained the structure of collagen layer surrounding hair follicle on days 2 and 5 compared to control (see Supplementary) and lymphatic vessel dilation and hyperkeratosis on days 7 and 14 were severely observed in control. When the thickness of collagen layer in the edematous granulated tissue was measured, the YIGSR treatment had a significantly 3.2 fold thinner collagen layer than the saline-treated edematous tail (106.2 vs 343.5 μm, *P* < 0.03, day 14) (Fig. 3D).

### 3.4 YIGSR treatment enhances keratinocyte cell-cell adhesion, reducing TGFβ, SM22α and β-catenin expression

The transmission electron-microscopic studies revealed morphologically, cell-cell adhesion at basal membrane in the saline-treated lymphedema differed significantly from cells contained in the YIGSR-treated lymphedema (Fig. 4A). In control, keratinocytes were in contact with each other intercellularly through adherens junctions and the intercellular spaces between keratinocytes was observed (Fig. 4B, Cell-cell adherence). By contrast, in YIGSR-treated group, keratinocytes adhered tightly each other on all aspect other than desmosome. Also, the morphology of lymphatic vessel and blood vessels differed (Fig. 4B, Lymphatic and Blood vessel). In control, lymphatic and vascular endothelial cell was damaged severely, and the contents exuded out from damaged both vessels. But, the structure of lymphatic and blood vessel was keeping in YIGSR-treated group. The state of damaged cell was totally more severe in control than in YIGSR-treated group.

To assess the effect of YIGSR on TGFβ signaling, we conducted RT-qPCR on the edematous tissues to determine the longitudinal expression of TGFβ1 and TGFβ2 and the mesenchymal markers SM22α and β-catenin. TGFβ1 and TGFβ2 expression peaked on day 7 while SM22α and β-catenin expression peaked on days 5–7. Interestingly, these peak timepoints largely corresponded to the lymphedema peak timepoint (day 7) (Fig. 4C). YIGSR treatment significantly reduced the expression of all genes, especially on day 7 (*P* = 0.030, 0.021, 0.042, and 0.036, respectively. n = 6–7).

## 4 Discussion

At present, the treatments for lymphedema are limited to massage, compression garments, prophylactic antibiotic treatment to prevent cellulitis, and in severe cases lymphatic venous anastomosis or vascularized lymph node transplant surgery [20]. However, the conservative methods generally only relieve symptoms and the surgery is invasive. Implantation of adipose-derived regenerative cells that promote lymphangiogenesis may be a useful treatment option in the future but further studies are needed [21]. Thus, further research into therapies that can prevent, ameliorate, or cure lymphedema is warranted. The present study showed that injections with YIGSR peptide, which is a component of laminin that binds to 67LR, significantly reduced the lymphedematous swelling in the mouse tail lymphedema model. Moreover, we noted that this associated with marked amelioration of the histological and TEM features of the lymphedema, including lymphatic vessel dilatation and damage.

These findings suggest that laminin, which is a key component of the basement membrane that anchors endothelial cells in the lining of vessels, plays an important role in lymphedema. We elucidated how expression and distinct distribution of laminin alter in lymphedema. In this mouse tail lymphedema model, we examined edematous tissues within 1 cm of the resection site. Binary imaging of immunohistochemistry appeared to localize laminin to sites of edema exacerbation during the course of lymphedema. Indeed, Kim et al. reported that over-expression of laminin was observed during vasogenic edema and correlates to recovery of endothelial barrier [22]. Quite recently, in vitro study on lymphangiogenesis revealed that laminin synthesis contributes to lymphatic basement membrane remodeling [23]. However, due to the poor quantification of our binary images, we examined the expression of laminin protein and found no time-dependent change in our edematous tissues. On the other hand, the time course of 67LR expression after the creation of lymphedema changed drastically to correlate with the tail swelling (Lymphedema in Fig. 1C and Saline in Fig. 2C). 67LR is expressed by the proliferating vasculature during neovascularisation, when achieved contact inhibition, 67LR expression is reduced to comparatively low levels [24]. 67LR has been shown to be overexpressed on the cell surface of various normal [19,25] and tumor [[26], [27], [28], [29], [30]] undifferentiated/proliferative cells in vitro and correlates with tumor growth and proliferation in vivo [31,32]. This notion is further supported by (i) our RT-qPCR analysis showing that 67LR, the laminin receptor, was upregulated in the lymphedematous tissue, and (ii) multiple in vitro studies that show endothelial cells adhere particularly strongly to YIGSR-bearing cell-adhesive materials and that this augments their formation of new vessels [[33], [34], [35], [36], [37], [38]].

**Fig. 5:**
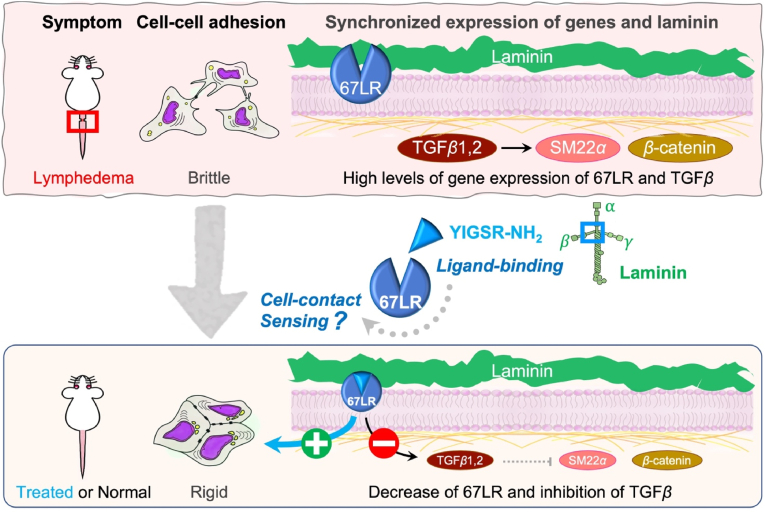
Putative mechanism by which early YIGSR treatment attenuates lymphedema. Putative mechanism may be explained by the assumption that cells in lymphedema are not completely contact inhibited whereas membrane-bound 67LR acts as a cell contact sensor, which substitutes for ligand binding. When 67LR senses directly or indirectly cell contact, it accelerates the formation of tight cell-cell adherence and 67LR itself decreases. It looks as if contact inhibition is pseudo simulated and the cell cycle is changed drastically. In addition, YIGSR is supposed to decrease TGFβ which negatively regulates lymphatic vessel regeneration. We suppose that suppression of excessive wound healing by downregulation of TGFβ leads to preventing from excessive inflammation and makes extracellular environment prompt proper wound healing.

Our study showed for the first time that treatment with YIGSR, the laminin peptide to which 67LR binds, reduced lymphedema, and that this associated with much more intercellular adhesion of keratinocytes [39,40]. These effects were appeared to be mediated by the binding of YIGSR to 67LR since blocking 67LR function with the anti-67LR antibody MLuC5 had the reverse effect, namely, exacerbated lymphedema. The precise molecular mechanism by which the early (day 0–3) YIGSR treatment protected the lymphatic system in the mouse tail is unclear. However, one possibility is that the binding of YIGSR to 67LR on epithelial and endothelial cells causes the intercellular adhesion as a cell survival factor. It is presumed that 67LR is highly expressed by detecting detachment, in which cell-cell adhesion is severely disrupted by edema. Conversely, 67LR senses cell-contact and decreases its expression after healing (Fig. 5). This is supported by studies on 67LR-overexpressing cancer cells: 67LR activation enhances cell-cell adhesion of cancer cells (creating pseudo-contact inhibition) to inhibit tumor growth and metastasis [[41], [42], [43], [44]]. YIGSR inhibits the colonization of the lung melanoma cells in mice [45]. Furthermore, YIGSR (1 mg/mouse)-injected tumor model mice lived for more than 2 months with a survival rate of 50% whilst the control animals died sooner than the YIGSR-treated animals, with 50% of the animals dead by day 18 [46]. Thus, YIGSR treatment would allow keratinocytes and endothelial cells to seal their intercellular spaces and restore the barrier function of the basement membrane [47,48].

YIGSR treatment may also reduce lymphedema more indirectly by suppressing TGFβ expression in the lymphedematous skin. This effect of YIGSR is consistent with previous studies showing that the inhibition of TGFβ with other treatments (e.g. anti-TGFβ antibody) accelerates lymphatic regeneration [[49], [50], [51], [52]]. It was notable that not only 67LR expression but also TGFβ expression peaked at day 5–7 before dropping sharply, while the lymphedema peaked at day 7 and then slowly improved. This suggests that early intervention targeting the lamininβ-67LR-TGFβ axis soon after the lymphedema trigger (e.g. surgery and/or radiation) could help prevent the onset of lymphedema. Previous study demonstrated that TGFβ-Induced Epithelial-Mesenchymal Transition (EMT) triggers the loss of cell-cell adhesive junctions [53]. Therefore, it was presumed that YIGSR-induced suppression of TGFβ resulted in decreased the expression levels of SM22 α and β-catenin, leading to block EMT [54,55]. TGFβ2-treated HDLECs increased expression of SM22α, a mesenchymal cell marker accompanied by increased cell motility and vascular permeability, suggesting HDLECs to undergo EndMT [56].

Our histological and TEM analyses also showed that lymphedema associated with epidermal thickening and hyperkeratosis. This is a well-known feature of lymphedema: indeed, epidermal thickening correlates with lymphedema severity in human patients [57]. These epidermal features are likely to be secondary to the fluid built up in lymphedema since volume-reducing surgery can reverse them [58]. Notably, we also found that the keratinocytes displayed loss of adherens junctions, and that all epidermal changes were greatly attenuated by YIGSR injection.

In conclusion, the protective effects of YIGSR on the epithelial barrier function and cell-cell contact were primarily accomplished by binding to 67LR, improving lymphedema also accompanied with the reduction in TGFβ expression levels. Further studies elucidating the underlying mechanisms may help reveal therapeutic targets for lymphedema.

## CRediT authorship contribution statement

Yuki Sakae: Methodology, Validation, Formal analysis, Writing - Original Draft.

Hiroya Takada: Conceptualization, Investigation, Writing - Original Draft, Writing - Review & Editing, Supervision, Funding acquisition.

Mayuri Nakajima: Formal analysis.

Shizuko Ichinose: Formal analysis, Investigation.

Atsushi Sakai: Formal analysis, Investigation, Writing - Review & Editing, Funding acquisition.

Rei Ogawa: Project administration, Funding acquisition.

## Funding information

This research was partially supported by 10.13039/100009619AMED (JNC: 9010005023796) under Grant Numbers JP22gm0810006 and JP23hma322006.

## Declaration of competing interest

The authors declare the following financial interests/personal relationships which may be considered as potential competing interests: Hiroya TAKADA reports financial support was provided by Nippon Medical School. Hiroya Takada reports a relationship with 10.13039/100009619Japan Agency for Medical Research and Development that includes: funding grants. Atsushi Sakai reports a relationship with 10.13039/100009619Japan Agency for Medical Research and Development that includes: funding grants. Rei Ogawa reports a relationship with 10.13039/100009619Japan Agency for Medical Research and Development that includes: funding grants. The Department of Anti-Aging and Preventive Medicine of Nippon Medical School is financially supported by donations from Angfa Co. Ltd., Tokyo, Japan.

## Data Availability

No data was used for the research described in the article.

## References

[1] Sugiura Y., Usui M., Miyata M. (2017). The suppressive effect of the lipophilic fraction of Eisenia arborea on ear swelling in mice. J. Natl. Fish. Univ..

[2] Massia S.P., Rao S.S., Hubbell J.A. (1993). Covalently immobilized laminin peptide Tyr-Ile-Gly-Ser-Arg (YIGSR) supports cell spreading and co-localization of the 67-kilodalton laminin receptor with alpha-actinin and vinculin. J. Biol. Chem..

[3] Hsu J.F., Yu R.P., Stanton E.W., Wang J., Wong A.K. (2021). Current advancements in animal models of postsurgical lymphedema: a systematic review. Adv. Wound Care.

[4] Hassanein A.H., Sinha M., Neumann C.R., Mohan G., Khan I., Sen C.K. (2021). A murine tail lymphedema model. J. Vis. Exp..

[5] Zhou C., Su W., Han H., Li N., Ma G., Cui L. (2020). Mouse tail models of secondary lymphedema: fibrosis gradually worsens and is irreversible. Int. J. Clin. Exp. Pathol..

[6] Ly C.L., Kataru R.P., Mehrara B.J. (2017). Inflammatory manifestations of lymphedema. Int. J. Mol. Sci..

[7] Frueh F.S., Gousopoulos E., Rezaeian F., Menger M.D., Lindenblatt N., Giovanoli P. (2016). Animal models in surgical lymphedema research--a systematic review. J. Surg. Res..

[8] Ghanta S., Cuzzone D.A., Torrisi J.S., Albano N.J., Joseph W.J., Savetsky I.L., Gardenier J.C., Chang D., Zampell J.C., Mehrara B.J. (2015). Regulation of inflammation and fibrosis by macrophages in lymphedema. Am. J. Physiol. Heart Circ. Physiol..

[9] Zampell J.C., Yan A., Elhadad S., Avraham T., Weitman E., Mehrara B.J. (2012). CD4(+) cells regulate fibrosis and lymphangiogenesis in response to lymphatic fluid stasis. PLoS One.

[10] Tabibiazar R., Cheung L.J., Han J., Swanson J., Beilhack A., An A., Dadras S.S., Rockson N., Joshi S., Wagner R., Rockson S.G. (2006). Inflammatory manifestations of experimental lymphatic insufficiency. PLoS Med..

[11] Lee J.H., Kishikawa M., Kumazoe M., Yamada K., Tachibana H. (2010). Vitamin A enhances antitumor effect of a green tea polyphenol on melanoma by upregulating the polyphenol sensing molecule 67-kDa laminin receptor. PLoS One.

[12] Di Russo J., Luik A.L., Yousif L., Budny S., Oberleithner H., Hofschröer V., Klingauf J., van Bavel E., Bakker E.N., Hellstrand P., Bhattachariya A., Albinsson S., Pincet F., Hallmann R., Sorokin L.M. (2017). Endothelial basement membrane laminin 511 is essential for shear stress response. EMBO J..

[13] Miner J.H., Li C., Mudd J.L., Go G., Sutherland A.E. (2004). Compositional and structural requirements for laminin and basement membranes during mouse embryo implantation and gastrulation. Development.

[14] Park H., Choi S.H., Kong M.J., Kang T.C. (2019). Dysfunction of 67-kDa laminin receptor disrupts BBB integrity via impaired dystrophin/AQP4 complex and p38 MAPK/VEGF activation following status epilepticus. Front. Cell. Neurosci..

[15] Kim J.E., Park H., Lee J.E., Kang T.C. (2020). Blockade of 67-kDa laminin receptor facilitates AQP4 down-regulation and BBB disruption via ERK1/2-and p38 MAPK-Mediated PI3K/AKT Activations. Cells.

[16] Li J., Ye L., Wang X., Liu J., Wang Y., Zhou Y., Ho W. (2012). (−)-Epigallocatechin gallate inhibits endotoxin- induced expression of inflammatory cytokines in human cerebral microvascular endothelial cells. J. Neuroinflammation.

[17] Lee H.S., Jun J.H., Jung E.H., Koo B.A., Kim Y.S. (2014). Epigalloccatechin-3-gallate Inhibits ocular neovascularization and vascular permeability in human retinal pigment epithelial and human retinal microvascular endothelial cells via suppression of MMP-9 and VEGF activation. Molecules.

[18] Graf J., Ogle R.C., Robey F.A., Sasaki M., Martin G.R., Yamada Y., Kleinman H.K. (1987). A pentapeptide from the laminin B1 chain mediates cell adhesion and binds the 67,000 laminin receptor. Biochemistry.

[19] Khalfaoui T., Groulx J.F., Sabra G., GuezGuez A., Basora N., Vermette P., Beaulie J.F. (2013). Laminin receptor 37/67LR regulates adhesion and proliferation of normal human intestinal epithelial cells. PLoS One.

[20] Kayıran O., Tane K., Soran A. (2017). Lymphedema: from diagnosis to treatment. Turk. J. Surg..

[21] Forte A.J., Boczar D., Sarabia-Estrada R., Huayllani M.T., Avila F.R., Torres R.A., Guliyeva G., Aung T., Quiñones-Hinojosa A. (2021). Use of adipose-derived stem cells in lymphatic tissue engineering and regeneration. Arch Plast Surg.

[22] Kim Y.J., Kim J.Y., Ko A.R., Kang T.C. (2014). Over-expression of laminin correlates to recovery of vasogenic edema following status epilepticus. Neuroscience.

[23] Jia W., He W., Wang G., Goldman J., Zhao F. (2022). Enhancement of lymphangiogenesis by human mesenchymal stem cell sheet. Adv. Healthcare Mater..

[24] Donaldson E.A., McKenna D.J., McMullen C.B., Scott W.N., Stitt A.W., Nelson J. (2000). The expression of membrane-associated 67-kDa laminin receptor (67LR) is modulated in vitro by cell-contact inhibition. Mol. Cell Biol. Res. Commun..

[25] Montuori N., Pesapane A., Giudice V., Serio B., Rossi F.W., De Paulis A., Selleri C. (2016). 67 kDa laminin receptor (67LR) in normal and neoplastic hematopoietic cells: is its targeting a feasible approach?. Transl. Med. UniSa..

[26] al-Saleh W., Delvenne P., van den Brule F.A., Menard S., Boniver J., Castronovo V. (1997). Expression of the 67 KD laminin receptor in human cervical preneoplastic and neoplastic squamous epithelial lesions: an immunohistochemical study. J. Pathol..

[27] Viacava P., Naccarato A.G., Collecchi P., Ménard S., Castronovo V., Bevilacqua G. (1997). The spectrum of 67-kD laminin receptor expression in breast carcinoma progression. J. Pathol..

[28] Sanjuan X., Fernández P.L., Miquel R., Muñoz J., Castronovo V., Ménard S., Palacín A., Cardesa A., Campo E. (1996). Overexpression of the 67-kD laminin receptor correlates with tumor progression in human colorectal carcinoma. J. Pathol..

[29] Givant-Horwitz V., Davidson B., Reich R. (2004). Laminin-induced signaling in tumor cells: the role of the M(r) 67,000 laminin receptor. Cancer Res..

[30] Pesapane A., Ragno P., Selleri C., Montuori N. (2017). Recent advances in the function of the 67 kDa laminin receptor and its targeting for personalized therapy in cancer. Curr. Pharmaceut. Des..

[31] Scheiman J., Tseng J.C., Zheng Y., Meruelo D. (2010). Multiple functions of the 37/67-kd laminin receptor make it a suitable target for novel cancer gene therapy. Mol. Ther..

[32] Kumazoe M., Sugihara K., Tsukamoto S., Huang Y., Tsurudome Y., Suzuki T., Suemasu Y., Ueda N., Yamashita S., Kim Y., Yamada K., Tachibana H. (2013). 67-kDa laminin receptor increases cGMP to induce cancer-selective apoptosis. J. Clin. Invest..

[33] Grant D.S., Tashiro K., Segui-Real B., Yamada Y., Martin G.R., Kleinman H.K. (1989). Two different laminin domains mediate the differentiation of human endothelial cells into capillary-like structures in vitro. Cell.

[34] Massia S.P., Hubbell J.A. (1991). Human endothelial cell interactions with surface-coupled adhesion peptides on a nonadhesive glass substrate and two polymeric biomaterials. J. Biomed. Mater. Res..

[35] Jun H., West J.L. (2004). Development of a YIGSR peptide-modified polyurethaneurea to enhance endothelialization. J. Biomater. Sci. Polym. Ed..

[36] Jun H., West J.L. (2005). Modification of polyurethaneurea with PEG and YIGSR peptide to enhance endothelialization without platelet adhesion. J. Biomed. Mater. Res. B Appl. Biomater..

[37] Ali S., Saik J.E., Gould D.J., Dickinson M.E., West J.L. (2013). Immobilization of cell-adhesive laminin peptides in degradable PEGDA hydrogels influences endothelial cell tubulogenesis, biores. Open Access.

[38] Çevik Z.B.Y., Ördek A., Karaman O. (2022). Regulatory effects of laminin derived peptide on microtissue formation for tissue engineered scaffold-free constructs. Eur. J. For. Res..

[39] Cavalieri S., Rotoli M., Feliciani C., Amerio P. (2005). Expression of the high-affinity laminin receptor (67 kDa) in normal human skin and appendages. Int. J. Immunopathol. Pharmacol..

[40] Balasubramanian S., Sturniolo M.T., Dubyak G.R., Eckert R.L. (2005). Human epidermal keratinocytes undergo (-)-epigallocatechin-3-gallate-dependent differentiation but not apoptosis. Carcinogenesis.

[41] Yu H.N., Zhang L.C., Yang J.G., Das U.N., Shen S.R. (2009). Effect of laminin tyrosine-isoleucine-glycine-serine-arginine peptide on the growth of human prostate cancer (PC-3) cells in vitro. Eur. J. Pharmacol..

[42] Zhou L., Yang F., Li G., Huang J., Liu Y., Zhang Q., Tang Q., Hu C., Zhang R. (2018). Coptisine induces apoptosis in human hepatoma cells through activating 67-kDa laminin receptor/cGMP signaling. Front. Pharmacol..

[43] Le C.T., Leenders W.P.J., Molenaar R.J., van Noorden C.J.F. (2018). Effects of the green tea polyphenol epigallocatechin-3-gallate on glioma: a critical evaluation of the literature. Nutr. Cancer.

[44] Kumazoe M., Hiroi S., Tanimoto Y., Miyakawa J., Yamanouchi M., Suemasu Y., Yoshitomi R., Murata M., Fujimura Y., Takahashi T., Tanaka H., Tachibana H. (2020). Cancer cell selective probe by mimicking EGCG. Biochem. Biophys. Res. Commun..

[45] Iwamoto Y., Robey F.A., Graf J., Sasaki M., Kleinman H.K., Yamada Y., Martin G.R. (1987). YIGSR, a synthetic laminin pentapeptide, inhibits experimental metastasis formation. Science.

[46] Kanemoto T., Reich R., Royce L., Greatorex D., Adler S.H., Shiraishi N., Martin G.R., Yamada Y., Kleinman H.K. (1990). Identification of an amino acid sequence from the laminin A chain that stimulates metastasis and collagenase IV production. Proc. Natl. Acad. Sci. USA.

[47] Yoon J.H., Kim J., Lee H., Kim S.Y., Jang H.H., Ryu S.H., Kim B.J., Lee T.G. (2012). Laminin peptide YIGSR induces collagen synthesis in Hs27 human dermal fibroblasts. Biochem. Biophys. Res. Commun..

[48] Kim Y.Y., Li H., Song Y.S., Jeong H.S., Yun H.Y., Baek K.J., Kwon N.S., Shin Y.K., Park K.C., Kim D.S. (2018). Laminin peptide YIGSR enhances epidermal development of skin equivalents. J. Tissue Viability.

[49] Avraham T., Daluvoy S., Zampell J., Yan A., Haviv Y.S., Rockson S.G., Mehrara B.J. (2010). Blockade of transforming growth factor-β1 accelerates lymphatic regeneration during wound repair. Am. J. Pathol..

[50] Baik J.E., Park H.J., Kataru R.P., Savetsky I.L., Ly C.L., Shin J., Encarnacion E.M., Cavali M.R., Klang M.G., Riedel E., Coriddi M., Dayan J.H., Mehrara B.J. (2022). TGF-β1 mediates pathologic changes of secondary lymphedema by promoting fibrosis and inflammation. Clin. Transl. Med..

[51] Clavin N.W., Avraham T., Fernandez J., Daluvoy S.V., Soares M.A., Chaudhry A., Mehrara B.J. (2008). TGF-beta1 is a negative regulator of lymphatic regeneration during wound repair. Am. J. Physiol. Heart Circ. Physiol..

[52] Fukasawa K., Hanada K., Ichikawa K., Hirashima M., Takagi T., Itoh S., Watabe T., Itoh F. (2021). Endothelial-specific depletion of TGF-β signaling affects lymphatic function. Inflamm. Regen..

[53] Gonzalez D.M., Medici D. (2014). Signaling mechanisms of the epithelial-mesenchymal transition. Sci. Signal..

[54] Zhou S. (2011). TGF-β regulates β-catenin signaling and osteoblast differentiation in human mesenchymal stem cells. J. Cell. Biochem..

[55] Zhou B., Liu Y., Kahn M., Ann D.K., Han A., Wang H., Nguyen C., Flodby P., Zhong Q., Krishnaveni M.S., Liebler J.M., Minoo P., Crandall E.D., Borok Z. (2012). Interactions between β-catenin and transforming growth factor-β signaling pathways mediate epithelial-mesenchymal transition and are dependent on the transcriptional co-activator cAMP-response element-binding protein (CREB)-binding protein (CBP). J. Biol. Chem..

[56] Yoshimatsu Y., Kimuro S., Pauty J., Takagaki K., Nomiyama S., Inagawa A., Maeda K., Podyma-Inoue K.A., Kajiya K., Matsunaga Y.T., Watabe T. (2020). TGF-beta and TNF-alpha cooperatively induce mesenchymal transition of lymphatic endothelial cells via activation of Activin signals. PLoS One.

[57] Wolf S., von Atzigen J., Kaiser B., Grünherz L., Kim B.-S., Giovanoli P., Lindenblatt N., Gousopoulos E. (2022). Is lymphedema a systemic disease? A paired molecular and histological analysis of the affected and unaffected tissue in lymphedema patients. Biomolecules.

[58] Torrisi J.S., Joseph W.J., Ghanta S., Cuzzone D.A., Albano N.J., Savetsky I.L., Gardenier J.C., Skoracki R., Chang D., Mehrara B.J. (2015). Lymphaticovenous bypass decreases pathologic skin changes in upper extremity breast cancer-related lymphedema. Lymphatic Res. Biol..

