# Supplementary material for: Treatment with YIGSR peptide ameliorates mouse tail lymphedema by 67 kDa laminin receptor (67LR)-dependent cell-cell adhesion
Source: Biochem Biophys Rep. 2023 Jul 20;35:101514. doi: 10.1016/j.bbrep.2023.101514 (PMC10372372; doi:10.1016/j.bbrep.2023.101514)
Supplement: Multimedia component 1 [file mmc1.docx]

*Short communication*

**Supplementary**

**Table S1.** List of qPCR primer pairs used to validate gene expression data.

## Fig. S1. Effect of YIGSR injections on lymphedemous tail histology (H&E staining).

(A) Representative tail cross-sections of the YIGSR- or saline-treated mice. (B) Zoom-in images of the areas in (A).

## Fig. S2. Effect of YIGSR injections on lymphedemous tail histology (Masson‘s Trichrome staining).

(A) Representative tail cross-sections of the YIGSR- or saline-treated mice. (B) Zoom-in images of the areas in (A).

## Fig. S3. RT-PCR of Prox1 and Lyve1 (biomarkers of lymphatic endothelial cells) expression in tail tissues from untreated operated and non-operated mice. Mean ± SEM are shown in the plots. **P*<0.03, ***P*<0.005, ****P*<0.001, as determined by Student’s one-sided *t*-test comparing operated mice to non-operated mice. List of qPCR primer pairs of Prox1 and Lyve1 used to validate gene expression data.

Table S1.

| 67LR | Forward | ATTCTCCCCTGCGCTATGTG |
| --- | --- | --- |
|  | Reverse | GATCAGGCATGACCTCCCAG |
| TGFβ1 | Forward | CTCTTGAGTCCCTCGCATCC |
|  | Reverse | GAGCGCTCTCTGAGATCCAA |
| TGFβ2 | Forward | CGAGGCGAGATTTGCAGGTA |
|  | Reverse | GCAGGAGATGTGGGGTCTTC |
| SM22α | Forward | TGGTTTATGAAGAAAGCCCAGGA |
|  | Reverse | GAAGGCCAATGACGTGCTTC |
| β-catenin | Forward | CTGGGACTCTGCACAACCTT |
|  | Reverse | CAGTGTCGTGATGGCGTAGA |

Fig. S1.

Fig. S2.

Fig. S3.

| Prox1 | Forward | TAAGCGAGAAGGCAGCAACA |
| --- | --- | --- |
|  | Reverse | AGACTTTGACCACCGTGTCC |
| Lyve1 | Forward | TTACCCCGTGTTGGACACTC |
|  | Reverse | GGCAGAAACAGGTGTTGTGG |
