# Supplementary material for: Treatment with YIGSR peptide ameliorates mouse tail lymphedema by 67 kDa laminin receptor (67LR)-dependent cell-cell adhesion
Source: Biochem Biophys Rep. 2023 Jul 20;35:101514. doi: 10.1016/j.bbrep.2023.101514 (PMC10372372; doi:10.1016/j.bbrep.2023.101514)

**Figure 1B. Original western blot.**

Expression of **laminin protein**

C3 and L3 were applied to Figure 1B (C: Control; L, L’: Lymphedema).


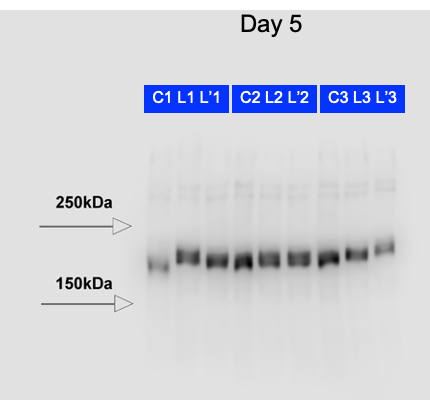


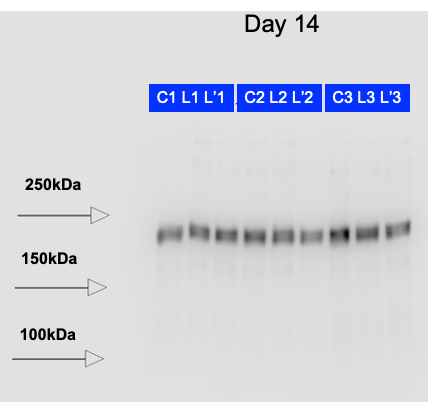

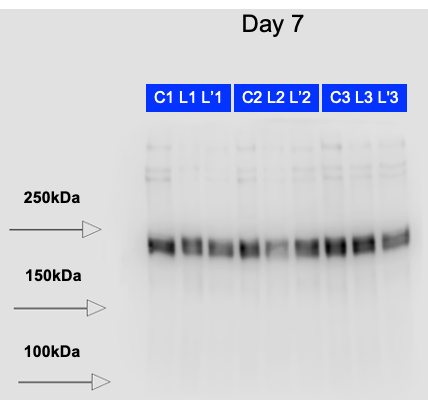

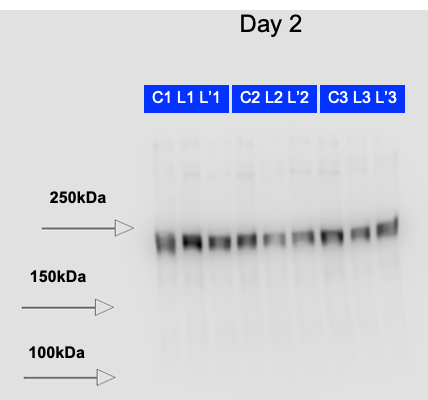


Expression of **β-tubulin** (approx. 50 kDa)

C3 and L3 were applied to Figure 1B (C: Control; L, L’: Lymphedema).


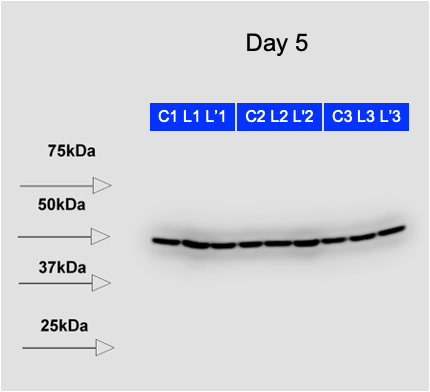

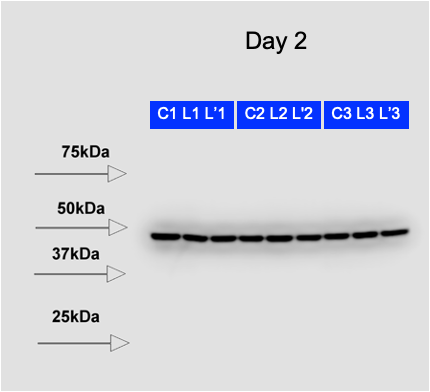


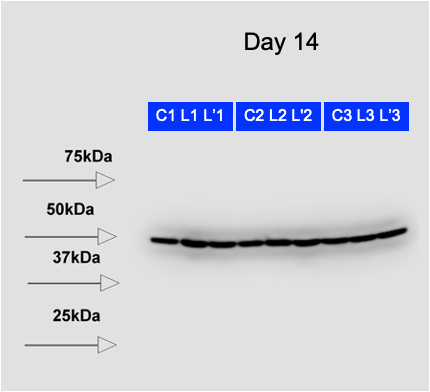

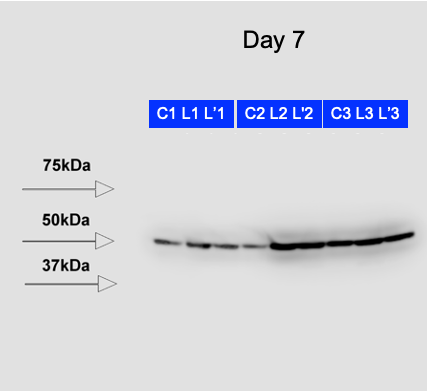

Supplement: Multimedia component 2 [file mmc2.docx]
